# On "Birth Palsy," with Illustrative Cases
*Paper read and cases shown at the Salisbury Branch Meeting of the British Medical Association, April 3rd, 1889.


**Published:** 1889-06

**Authors:** F. W. Jollye

**Affiliations:** Warminster


### Clinical Records.

---

### ON "BIRTH PALSY,"* WITH ILLUSTRATIVE CASES. By F. W. Jollye, M.R.C.S., L.R.C.P., Warminster.

The patients I shall bring before you to-day illustrate a class of cases which was first mentioned by Heine in 1840, and has since been described by Little,† Gowers,‡ M'Nutt,§ Gee,|| and Ross,¶ amongst others. Gowers calls these cases "Birth Palsies," which, as he points out, is much more correct than "Congenital" or "Obstetrical Palsies." The objections to the word "congenital" are these: (1) The word conceals a certain amount of ignorance as to their pathology; and (2) They are not begotten with the embryo, and therefore are not germ or congenital diseases such as syphilis and some forms of nervous affections, as, for example, pseudo-hypertrophic paralysis, and hereditary locomotor ataxy or Friedreich's disease. Another class of deformity is what is known as congenital talipes, which is due to malposition of the limbs in utero, and not, strictly speaking, to any congenital cause.

* Paper read and cases shown at the Salisbury Branch Meeting of the British Medical Association, April 3rd, 1889. † *Obstetrical Trans.*, 1862.

‡ *Diseases of the Nervous System*, vol. II.

§ *American Journal of the Medical Sciences*, 1885.

|| *St. Bartholomew's Hospital Reports*, 1877, 1880. ¶ *Brain*, vol. VI.

These cases of Birth Palsy do not belong to either of these classes, but form quite a distinct class, whose symptoms are due to injury received during the process of birth. They are not all strictly obstetrical; for the accoucheur does not inflict the injury in the most important and serious classes of these cases, although he might perhaps prevent their origin in some of them by timely interference in abnormal presentations, or by the application of the forceps in prolonged and difficult labours with head presenting.

Birth Palsies are divisible into two classes:

I. Peripheral or True Obstetrical Palsies.—Of this class, the common forms are:

*(a) Facial palsy*, from pressure of the blade of the forceps on the trunk of the seventh nerve after it has left the stylo-mastoid foramen and is crossing on to the lower jaw.

*(b) The "upper-arm type" of paralysis, or Erb's paralysis*, due to injury of the cord formed by the fourth, fifth, and sixth cervical nerves above the clavicle in breech presentations by pressure on this cord by the tip of the finger, or by the point of the traction-hook just in front of the trapeezius, causing paralysis of the deltoid, biceps, brachialis anticus and supinators, and often with these the infra- and supra-spinatus muscles. The muscles, although supplied directly by the following nerves: circumflex, musculo-cutaneous, musculo-spiral and supra-scapular, yet all indirectly are supplied, fully or partly, by the fourth, fifth, and sixth cervical nerves. The reason of this is, that all synergic or associated movements are related to special groups of nerve-cells situated near together in the grey matter of the spinal cord; and, therefore, injury to these cells, or to the motor roots springing from them, causes paresis or paralysis of associated muscles. As another example of associated movements in this neighbourhood, I may mention that the centres for the two parts of the pectoralis major are separate, and are associated—the clavicular with that of the serratus magnus, the costal with that of the latissimus dorsi.

*(c) Irregular paralysis of the arm,* from pressure of a fractured humerus or a dislocation at the shoulder-joint, or what is often mistaken for the latter—a separation of the upper epiphysis. The musculo-spiral is the nerve most often affected, then the ulnar, and very rarely the median.

As a rule all these peripheral forms recover perfectly in a few days or weeks. The muscles may show the reaction of degeneration during recovery, if this takes some time.

II. Cerebral Class.—The lesion in this division is a much more serious one: it may cause death soon after birth; or it may, by causing paralysis and spasm of the limbs, give rise to deformities which may last for years, or even for lifetime; or, lastly, it may cause deterioration of the mind, from simple weakness of intellect up to actual idiocy. Any of these symptoms may be associated with epilepsy.

The history of these cases is, shortly, as follows: There will be no history of any definite onset; but the mother will say that nothing was noticed the matter with the child until it was found that it did not walk at the usual time, and on closer examination that the legs were found to be more or less stiff. In the more severe cases immobility of the limbs is noted during the early weeks of life, either on one or both sides, or both legs; or occasionally only one limb, and that more often the arm. If the birth is enquired into, it is often found that the labour was difficult, or it was an abnormal presentation such as a breech, and that it may have been the first child. In many cases, as a proof of the difficulty of birth, you may be told that the child appeared dead for some minutes, and was only restored by artificial respiration; or it may have had a large hæmatoma of the scalp; or it may have been convulsed generally, or only on one side, followed by spasm, universal or local, during the first few hours or days of life. The convulsions are often erroneously ascribed by the parents as the cause of the rigidity and paralysis.

Out of 36 cases seen by Dr. Gowers, $\frac{1}{5}$ were breech presentations. Of the remainder, 17 out of the 24 in which this point was noted were first-born; and in the remaining multiparous ones, most of the previous labours had been difficult. All four limbs were affected in $\frac{2}{3}$ of the cases ($\frac{22}{36}$), trunk and neck-muscles in $\frac{1}{3}$ ($\frac{11}{36}$), limbs of one side only affected in $\frac{7}{34}$, and in rather fewer ($\frac{6}{34}$) the legs alone suffered in considerable degree.

*Symptoms.*—The noticeable feature of these cases is the paralysis, more or less, of some of the muscles, combined with spasm. If the child is able to walk, it will be noticed that the legs are moved very stiffly; that they are kept wide apart, so that he can more easily balance himself; and that he walks very often on his toes, the heel being drawn up. Their gait is very unsteady, and they easily fall. If the legs are examined, the feet will be found in a state of talipes equinus, or equino-varus, due to spasm of the calf-muscles; but this can almost always be overcome by pressure on the sole. The hamstrings, too, are often in a state of spasm, causing flexion of the knees; or the knees may be flexed at one time, and at another rigidly extended. Spasm of the adductors—a common symptom—is well seen in the girl N. This adductor spasm may be sufficient to cause one leg to cross over the other in walking, causing "cross-legged progression," and this latter symptom is more marked in cerebral than spinal cases. There may be choreic movements of the two feet. The knee-jerks are excessive, and rectus-clonus may be easily obtained; but, as a rule, there is no ankle-clonus—there is none in the cases exhibted—which is peculiar: but Gowers accounts for it in this way—but his is only theory—"that the muscle-reflex mechanism related to the calf-muscles has not received the functional development that must result from the ever-recurring sequence of tension and contraction involved in walking. The active contracture in the calf-muscles, which most cases present, is a serious hindrance to walking, even when the muscular power is sufficient." It must be remembered that it has now been almost conclusively proved that the "deep reflexes are not true reflexes at all, but that they are locally excited in the muscle through the medium of the tendon, the muscle having had its irritability increased to local stimulation reflexly by tension previously brought about in it by extension of the muscle."

In the arms, besides the weakness, there is usually inco-ordination from spasm, and often spontaneous movements: the latter may be slow like athetosis, or quick like chorea; and some of these cases have been described as cases of "congenital chorea." If the spasm is marked, it throws out of gear all the voluntary and co-ordinated movements. If the child is asked to pick up a pin, it will often be noticed that his wrists, and fingers at the metacarpo-phalangeal joints, are hyper-extended; and then the fingers are expanded, and after a time close upon it in a spasmodic manner. This is well seen in the boy. The elbow may be spasmodically flexed and extended, and sometimes strongly adducted.

In infancy there may be conspicuous difficulty in supporting the head; in some there may be retraction of the head and some lateral curvature of the spine from weakness of the spinal muscles. The face, when quiet, sometimes appears normal; but on articulation there may be marked spasm, as seen in the boy W. F. Strabismus, if present, is due to spasm, not to paralysis. Mental defects are met with in half the cases, according to Gowers: in some cases amounting to idiocy; but generally it is only enough to render the acquisition of speech later than usual, and it is often difficult to know whether the inability to speak is due to motor aphasia from implication of Broca's convolution, or to weakness of intellect. In many cases in which the legs only appear affected some deficiency in the arms may often be detected; but I can find none in the girl N.

Fits are a common symptom: they may cease after a few months, or develop into confirmed epilepsy, but not so frequently as after acquired hemiplegia of children. They generally involve the limb most affected by the paralysis. The occurrence of fits bears no relation to the severity of the limb-symptoms. The initial convulsions of birth seldom persist; but subsequently fits begin, usually towards the end of the first year of life.

The limbs may be small from disuse; but there is no marked muscular wasting, and on electrical examination there is no reaction-of-degeneration.

*Pathology.*—The head of a child in a difficult labour suffers from a considerable amount of mechanical congestion, which may lead to rupture of veins and extravasation of blood amongst the meninges of the brain. We can easily understand this liability to rupture of these veins when we remember that, contrary to the rule elsewhere, ascending arteries open into ascending veins, and these veins open into the longitudinal sinus in such a manner that the entering blood is opposed in direction to the current in the sinus, and in the latter are trabeculæ running across the lumen. All these combined must offer great hindrance to the flow of blood.

This extravasation of blood takes place, as far as we know at present, in two places; viz., on the vertex over the motor region and its immediate neighbourhood, and at the base round the medulla and cerebellum from rupture of the cerebellar arteries. The symptoms of the latter form—*i.e.* at the base—are doubtful; but in those cases in which there is retraction and weakness of the neck, as well as spasm of the limbs, there is most probably extravasation round the medulla as well as over the motor area.

Dr. M'Nutt thinks that when the presentation is natural the extravasation is chiefly at the base, and that when the head is born last the blood is chiefly over the convexity; but post-mortem cases are too few at present to settle this question.

When the hæmorrhage is on the vertex, it occurs usually symmetrically, and the clot is generally thickest over the motor area, and especially near the longitudinal sinus; and as the centre more especially representing the leg occupies the margin of the longitudinal fissure and the paracentral lobule on the median aspect, we can understand why the legs are often more severely affected than the other limbs, and also why both are usually affected, resembling paraplegia due to disease of the spinal cord. The chief difference in the state of the legs in paraplegia from its state in hemiplegia is, the much greater spasm in the former (paraplegia). This is easily explained by Broadbent's hypothesis, which is: "Movements are represented exclusively in the opposite hemisphere in proportion as they are unilateral or voluntary (as in the arm); in both hemispheres in proportion as they are bilateral or automatic (as in the legs)." In other words, either hemisphere can excite bilateral movements, but only the opposite one can excite the unilateral movements; and, therefore, in hemiplegia innervation from the hemisphere of the same side acting through the lower spinal centres, ensures some recovery of power and moderation of the spasm. But if both centres are implicated, as is usually the case with the legs, compensation cannot occur; and bilateral degeneration, or perhaps arrest of development of the pyramidal tracts, occur, producing the same results as if due to primary disease of those tracts in the cord.

If the hæmorrhage spreads downwards, it will next affect the centre chiefly representing the arm, and the last to be involved will be the face-centre and the centre for speech. It follows that the pressure on the arm- and face-centres being less than that on the leg-centre, the former parts will have most chance of recovery, both by restoration of the damaged cells to a slight extent, but more probably by compensation taking place on the same and opposite sides of the brain. For the same reason, we are more likely to get choreic movements in the upper limbs; for, as Hughlings Jackson says, "In chorea we find persisting paralysis, with over-movements of the same parts: what has been called loss, or defect of voluntary co-ordination in the disease, is indirect evidence of some paralysis; the involuntary disorder of co-ordination is owing to over-activity of movements left."

Epilepsy from birth may, no doubt, be the only symptom of a very small but similar lesion.

The exact part of the cortex, injury of which will cause the mental symptoms found in these cases, is doubtful, owing to the difficulty of finding limited lesions not causing other symptoms as well. From the experiments of Ferrier, it would seem that the præfrontal lobes were especially concerned in mental processes; but Rosenthal, Bastian, and Hughlings Jackson think that psychical disturbances are incomparably more frequent, owing to destruction of the occipital lobes. At any rate, it seems certain that bilateral affections of the cerebral hemispheres—such as we see in the severe cases of birth palsies—are especially liable to produce mental defects, most probably due to some microscopical changes of the cortex both in front of and behind the so-called motor region.

The defects of speech may be divided into (1) motor aphasia or aphemia, and (2) where it is due to weakness of intellect. The boy W. F. belongs to Class 1; for his intelligence seems to be very fairly good, for he makes himself understood by filling up the defects in speech by gestures. The reason that his speech is so long in recovering is due, I think, to the fact that both the left and right lower frontal convolutions were damaged; for if only the left one had been affected, the right one would, being normal, have performed the work usually done by the left from the first.

The direct evidence we have that this is the correct pathology is the following:

(1) Children who have died very soon after birth, with the above symptoms of cerebral disturbance, have presented, on post mortem examination, symmetrical meningeal hæmorrhage, thickest over the motor area, and especially over the leg-centre.

(2) In post mortem examinations of children who have lived for some years, depression and shrinking of the motor areas on both sides have been found, more marked over the leg-centre, and gradually diminishing as you approach the area for the face. This shows that formerly there was pressure over this area, and, combined with the previous evidence, that it was exerted by extravasated blood which had been slowly absorbed.

PROGNOSIS.—Contrary to the general run of nervous cases, the prognosis in these cases is, on the whole, good. The symptoms tend to lessen gradually of their own accord as the child's age increases; and therefore we can always tell the parents, except in the very worst cases where there is actual idiocy, that the disease tends towards cure. With the latter exception, all the cases after a time stand, and then begin to walk. The little boy W. F. went about on his hands and knees until he was about eight years old, and now he can walk fairly well.

A great guide in the prognosis is the mental capacity, which we cannot gauge for two or three years after birth. It is important for two reasons: First, because the worse the defect of mind, so much the worse is the general prognosis; and secondly, if the child can concentrate his attention, and will deliberately try and help his muscles to work, the greater is the prospect of motor improvement: so you will see that the future power of progression depends a good deal on the mental defect; but the reverse does not hold good, for a child's motor power may be very little affected, but yet his mental symptoms may almost amount to idiocy, and never improve, or but very little. In those cases of slight mental defect, the intellect will be later in developing than in other children.

The prognosis as to the cure of the epileptic fits must be guarded, and the result of treatment watched. The prognosis, according to Gowers, is not so good as in idiopathic epilepsy; but is better than in epilepsy following hemiplegia in children. The early commencement of the fits renders the prognosis worse than if they began after puberty.

The improvement in the muscular spasm and in co-ordination is very slow for the first few years of life; but when once they are able to stand and walk alone, their improvement is much more rapid; but in all severe cases there is always some spasm, producing a peculiar gait, left. The choreic movements, if severe, often improve markedly along with the other symptoms.

Diagnosis.—The most important distinctions from other cerebral diseases are:

(1) There is no history of any definite onset at any time after birth.

(2) The weakness and spasm of the limbs does not get gradually worse; but if not stationary, the tendency is always to improvement. If the child is over three years old, and is getting worse, you may be pretty certain that it is not a case of "birth palsy."

If the case is of the bilateral type, affecting both arms and legs, it will have to be diagnosed from—

*Tumour in the Pons*—usually tubercular or a glioma—may cause paralysis with inco-ordination, and sometimes with choreic movements. In these cases there is a history of definite onset and progressive deterioration, with perhaps optic neuritis, headache, and affections of sensation of the limbs and of the fifth nerve and other symptoms of tumour, to guide you.

If only the legs are involved, the arms and face escaping, it will have to be distinguished from *paraplegia from pressure on the cord.*

If it is a case of birth palsy, you will usually find some awkward movements of the hands (I cannot find any in the girl N.), and there is no history of definite onset. Primary lateral sclerosis is, I believe, unknown in children; paraplegia at this time of life being generally due to spinal caries, in which case there will be symptoms of nerve-root irritation, such as girdle and lightning pains, often with angular curvature and spinal tenderness, with loss of control over the bladder and rectum. In neither case will there be marked muscular wasting.

*Pseudo-hypertrophic paralysis* resembles this disease in that the child walks later than usual and in an awkward manner, the muscles are not wasted—some often enlarged—and liability to contraction of the calf-muscles. It is distinguished by the facts, that it is often hereditary, so that more than one child may be afflicted in the same family; it tends gradually to get worse; and there is often congenital absence of the sternal part of the pectoralis major, with its associated muscle, the latissimus dorsi, often combined with enlargement of the infra-spinati.

In birth palsy the contraction of the gastrocnemii can be overcome by steady pressure on the sole, as it depends on muscular spasm; while in hypertrophic paralysis there is structural shortening, often with enlargement of the belly of the muscle, which cannot be overcome. The knee-jerks and superficial reflexes in the latter are never increased: they may be normal, but more often they are diminished or lost; while in cases of birth palsy they are increased.

*From progressive muscular atrophy of children*—a not common disease—like the case whose photograph I hand round: the muscular wasting, accompanied or followed by paralysis without any active spasm, although there may be organic shortening of the calf-muscles secondary to paralysis of their opponents, as in the photograph, together with the history that the child walked well for a few years, and the diminution or abolition of the reflexes, will easily settle the question.

*From congenital talipes equinus or equino-varus* it is important to distinguish it, as what is good treatment in the one does no permanent good in the other. It must be remembered that in the true talipes, due to malposition of the feet in utero, there is permanent shortening of the muscle; while in the other case there is only active contraction with no real shortening, and therefore tenotomy can do no real good.

The history of the case, the exaggerated reflexes, ability to flex the ankle considerably by pressure on the sole, besides the affection of the muscles of the thigh and of the hands which may often be found, will easily distinguish the case of birth palsy.

*Acute spinal paralysis of infants* is recognised by its usually definite onset with pyrexia and vomiting, followed in the course of a few hours or days by signs of paralysis, which in the course of a few weeks partly recovers, that limb or part of limb not recovering exhibiting well-marked muscular wasting with the reaction of degeneration. If it occurs in a very young child, its onset may be readily overlooked, and the parents then often date the disease from birth; and as the anterior tibial muscles and the extensors of the knee are the muscles very often involved, the deformity produced resembles that found in birth palsy: but in the cases of spinal paralysis the muscles on the anterior surface of the leg and thigh will be more or less wasted, the reflexes will be much diminished or quite gone, pressure on the sole will not flex the ankle, and the other leg may be perfectly sound.

In those rare cases in which the disease spreads from the grey matter in the cervical enlargement to the lateral columns the legs will present the same spastic paralysis as in birth palsy, but there will be found muscular wasting in the hands and arms. If the transverse myelitis attacks the lumbar region, there will be the acute onset, followed by wasting of some of the muscles of the legs, to guide one, together with some slight or more severe anæsthesia of them and girdle-pain round the trunk at the level of the lesion, combined with bladder and rectal troubles. In none of these cases will there be the choreic movements and spastic inco-ordination so common in the hands of the cases of birth palsy.

*In Friedreich's disease, or hereditary locomotor ataxy*, there are, as the name implies, other members of the family very often affected. The child walks well for some years, the disease generally manifesting itself about the sixth or seventh year, sometimes before, with ataxy of the legs, which in the course of a few years spreads to the arms, head, and neck. The knee-jerk and superficial reflexes are lessened, except in one recorded case, and there is often nystagmus. There is always a tendency to grow worse, although it may not appear to shorten life; but they usually die between five and twenty years after the onset.

If the case is hemiplegic in character, it will have to be distinguished from *acquired hemiplegia of children*, which results either from thrombosis of a surface-vein following some weakening illness, as one of the infectious fevers, or occasionally from embolism, or, according to Strümpell, from an acute polic encephalitis. This latter affection is chiefly found during the first five years of life; but it comes on suddenly with convulsions, and the leg may after a time get well, leaving only the arm affected, which alone is afterwards convulsed if convulsions recur. In birth palsy the symptoms, if carefully examined, are rarely hemiplegic: one side may be more affected than the other; but if you examine the other or supposed sound side, you will find some slight disturbance of the motor power present, especially in the finer movements of the hands and feet.

*Treatment.*—In all the destructive lesions of the brain from hæmorrhage, tumour, or embolism there are usually two directly opposite states to take into consideration in any treatment adopted. There is what is known as the negative functional state, or paralysis due to actual destruction of nerve-tissue, and which cannot be influenced so much by drugs, as strychnia, as is sometimes supposed; for, as Hughlings Jackson has said, "it is like treating a hole in the brain." Then there is the diametrically opposite state—the positive, super-positive, or over-functional state—met with in chorea, epilepsy, and muscular spasm. The two states may co-exist, and they do so in the cases before us. Some elements of the motor nervous arrangements have lost function, as seen by the weakness of the limbs; whilst other elements of the same set are in a state of over-function, as shown by the spasm in all the cases and by the epilepsy in the boy; and in fact it is this state of over-function of the remaining nervous elements which is the cause of the most apparent symptoms, and which is the great hindrance to the acquirement of the proper use of the limbs. From this preliminary statement it will be seen that all drugs classed as nervine tonics cannot possibly do much good, and may do great harm; for they cannot overcome the negative functional state or paralysis, and they are very likely to increase the super-positive functional one or spasm, which we want carefully to avoid. We must therefore devote all active treatment in trying to allay the spasm, which may be done by gentle massage of the muscles, and by gymnastic exercises to increase the power and rhythmical working of the various groups of muscles. To prevent increasing deformity of the feet and legs likely to ensue from the child walking on the edge of the feet, suitable instruments may be used, provided that with their help the child is enabled to stand and have more use of his limbs than he had without them. Tenotomy, as I have said can do no permanent good; and the same may be said of electricity, according to Gowers: after both the child improves, but that is only from lapse of time and not in any way the result of treatment.

We have not yet arrived at the stage of trephining new-born infants with the intention of removing the clot: and, considering the size of the clot, it is doubtful if it could be extracted; and if it was, whether the child could possibly survive the operation.

A good deal might be done in cases with motor asphasia or aphemia, like the boy W. F., to increase their power of articulation by showing them how to place their tongues and lips for utterance, and then making them emit a musical or non-musical breath according to the sound required.

The only other feature in the treatment necessary to speak of is that of the epileptic fits which are met with in some cases; in what proportion of cases I do not know. The cause of these fits is a "discharging lesion" composed of cells whose nutrition is abnormal, and whose power of resistance to the explosion of nerve-force therefore is diminished. What we have to aim at in treatment is, by the administration of drugs, to increase the stability of these cells, and so, by prolonging the intervals between the fits or arresting them altogether, to break them so to speak of their vicious habit. Treatment should be begun as early as possible for the shorter the duration of the disease, the greater is their chance of arrest. Bromide of potassium certainly diminishes the frequency of the fits in the boy W. F., and I think very likely might cure him if he would only persevere regularly in treatment. Perhaps the fits naturally decrease in frequency as the motor symptoms improve. Twice in the last year he has been in "status epilepticus" for several hours at a time. In passing I may remark that the "status epilepticus" requires very different treatment from the ordinary epileptiform convulsion. Stimulants and food may be required, and chloral-hydrate injected into the rectum gives the most permanent good results: but while this has time to act chloroform may be administered; but as a rule, according to Gowers, the latter does not give such a permanent result. In the first attack I saw him in, I injected twenty grains of chloral into the rectum with very good results. In the second attack Mr. Willcox administered chloroform, the effect of which had to be kept up several hours to prevent the return of the convulsions. Of course there is the possibility that the second attack may have been a severer one, but as far as I can judge the chloral gave the most satisfactory result. If drugs do arrest the fits, it is very important, according to Gowers, as in all cases of epilepsy, to continue them in undiminished doses for at least two years after the last fit, and then gradually reduce the dose for another year. If you let the fits return, you will have most probably to begin the cure again, just as you have if a congenital hernia reappears after some months of truss pressure.

Case 1.—E. N., a girl aged 8½ years. She is the eldest of four children, the remaining three being free from any deformity. The mother states that she was in labour seventeen hours, but was delivered without forceps, the head presenting. The child has never had any convulsions or illness.

The mother never noticed anything the matter with the child until she found that the latter could not walk at the usual time, and then the legs were found to be more or less stiff and the heel was drawn up.

She began to walk with help when she was four years old, and ever since she has gradually improved.

December, 1888.—She walks, with the help of a stick, as if her hips were stiff, rather shuffling along and taking short steps, the heel being rotated outwards on lifting it off the ground. She can only dorsally flex the feet to a right angle owing to the tension of the calf-muscles, but by pressure on the sole the foot may be still further flexed. The plantar arches are slightly increased, but there is no hyper-extension of the first phalanges or clawing of the toes. The hips can only be slightly flexed without previously flexing the knee to relax the hamstrings, and they cannot be separated very widely apart owing to spasm of the adductors. The knee-jerks are very brisk, but there is no ankle-clonus. There is no paralysis or spasm of any of the muscles of the hands, arms, or face. Her speech and intelligence are good, and she goes to school.

*Remarks.*—In this case of the paraplegic type of "birth palsy" the lesion was bilateral affecting that part of the brain especially representing the leg on each side of the longitudinal fissure. Contrary to the ordinary rule, I can find no affection of the arms. The slight increase of the plantar arches is due to the original weakness of the flexors of the ankle, followed by dropping of the foot in front of the transverse tarsal joint, with secondary contraction of the plantar fascia.

The diagnosis of this case from congenital talipes equinus or spastic paraplegia due to spinal cord disease, I have already pointed out.

Case 2.—W. F., a boy aged 11¾ years, is the youngest of eight children. The mother was subject to epileptic fits for twenty-five years before her death in 1887. The birth, as far as I can gather from the father, seems to have been natural, but the real facts are difficult to obtain.

When seven or eight months old he began to have epileptic fits, which have continued until the present time, although not so frequently during the last two years. When it was time for him to walk it was found that it was impossible; and if held up, only his toes touched the ground and the knees were flexed owing to contraction of the calf- and hamstring-muscles. It was also noticed that his hands were used very awkwardly, so that he had great difficulty in feeding himself. For several years he could only make inarticulate sounds, but understood what was said to him, and made others understand by gestures.

He has always been cleanly in his habits. Up to the middle of 1886 he used to get about the house on his hands and knees, but since then he began to stand and get about by steadying himself against objects as he went along.

June, 1888.—Walks very unsteadily by himself with the legs widely separated and rather rigid, and his feet extended, bearing the weight of the body on the balls of the toes, the heels not quite touching the ground. There is no marked wasting of any of the muscles of his arms or legs. The knee-jerks are very much exaggerated but there is no ankle-clonus. The ankle can be passively flexed very readily by upward pressure on the sole. The hamstrings are in a state of spasm, and so prevent him fully extending his knee, but there is no marked spasm of the adductors.

There are no spontaneous movements of his legs or arms.

In the arms there is slight spasm of the biceps, which is readily overcome. On telling him to hold his arms out straight you will see that the wrists are hyper-extended, as are also the phalanges. On being told to pick up a pin he hyper-extends his wrists and phalanges and separates his fingers, and then picks it up by slowly approximating the tips of his first finger and thumb, still in the extended position. He closes his fist slowly but firmly. There are no choreic movements of the hands on movement.

There is no spasm of the muscles of the back or neck.

There is no facial paralysis or spasm when at rest; but the muscles round the mouth and the muscles that close the jaws are thrown into spasm when he attempts to answer questions, the teeth being firmly clenched at that time. It is very difficult to understand what he says.

He is unable to protrude his tongue much beyond the incisor teeth when told to do so, nor has he ever been noticed to protrude it of his own accord.

In the epileptic fits in which I have seen him, rigidity of one or other side, the head and eyes deviating to the same side—that is, away from the side of the lesion,—have been the early symptoms.

March, 1889.—His symptoms have gradually been improving: his speech is decidedly more distinct; his walking is firmer, the heel coming nearer the ground. The epileptic fits continue as before—perhaps less frequently—happening more often when he is in bed than during the day.

*Remarks.*—This case is an example of the double hemiplegic class of "birth palsy," the lesion involving more or less the whole motor tract on both sides, including the centre for speech, but not affecting the occipital or præfrontal lobes, and so not causing any marked mental or sensory symptoms.

This case illustrates the long time a child may be unable to stand (eight years), and yet recover very useful walking-power by the age of 11. It also illustrates the general rule, that in recovery from hemiplegia those movements are first restored which are bilateral and most automatic; the unilateral or least automatic ones, such as the fine movements of the fingers and thumb, being the last to recover, just as they very often are the first to be affected in some chronic degenerative changes of the central nervous system — for example, progressive muscular atrophy and paralysis agitans,—both of which usually begin in the small muscles of the thumb and index-finger, the least automatic or most voluntary part of the least automatic limb—the arm.

